# Effect of Adding Fermented Proso Millet Bran Dietary Fiber on Micro-Structural, Physicochemical, and Digestive Properties of Gluten-Free Proso Millet-Based Dough and Cake

**DOI:** 10.3390/foods12152964

**Published:** 2023-08-05

**Authors:** Jing Xiao, Yinxia Li, Li Niu, Ronghui Chen, Jiayu Tang, Zongbo Tong, Chunxia Xiao

**Affiliations:** 1 College of Food Science and Engineering, Northwest A&F University, Yangling 712100, China; xiaojing12745@163.com (J.X.); lyx10712@163.com (Y.L.); niulikaoyan@163.com (L.N.); chenrh251003685@163.com (R.C.); tjy201816@163.com (J.T.); 2 College of Chemistry and Pharmacy, Northwest A&F University, Yangling 712100, China; cdslz212@ksu.edu

**Keywords:** proso millet flour, dietary fiber, cake, physicochemical quality, functional characteristics

## Abstract

The increasing demand for functional foods has pushed the food industry to produce fiber-enriched products. In this study, rheological, microstructural, physicochemical, and functional characteristics were investigated for whole proso millet dough and cake, fortified with fermented proso millet bran dietary fiber flour (F-DF). Results showed that proso millet flour is less absorbent and stable than the control group. Adding proso millet flour and F-DF reduced the elasticity of the dough and increased its hardness, but had no significant effect on viscosity, cohesion, and resilience. The microstructure analysis exhibited an unformed continuous network formation in proso millet dough. Analyses suggested that proso millet flour combined with the fermented dietary fiber group had significantly higher total phenol content (0.46 GAE mg/g), DPPH• scavenging activity (66.84%), and ABTS•+ scavenging activity (87.01%) than did the other group. In addition, F-DF led to a significant reduction in the predicted released glucose contents of reformulated cakes. In summary, cakes prepared with the involvement of whole proso millet flour and F-DF exhibited less adverse sensory impact and possessed the potential to decrease postprandial blood glucose levels resulting purely from cake consumption.

## 1. Introduction

Celiac disease (CD) is an immune-mediated, systemic disease [1]. In many countries, the number of newly diagnosed cases of CD has increased indexically over the past 30–40 years, and its etiology remains unclear [2]. The gluten-free diet remains the sole essential therapy for celiac disease, thus fueling a surge in demand for gluten-free products [3,4]. However, due to its purified starch-based composition, an unbalanced gluten-free diet is also linked to nutritional deficiencies, such as protein and dietary fiber insufficiency, as well as a high GI [3,5]. Therefore, the formulation of gluten-free foods requires the prudent selection of raw materials.

Proso millet is extensively cultivated in the arid and semiarid tropics of the world, including in Asia and Europe. Native to northern China, proso millet was one of the world’s earliest domesticated crops used to make liquor and various pastries [6]. Proso millet is gluten-free and nutrient-rich, with high levels of resistant starch and amylose [7]. In particular, proso millet-based products offer a lower GI than corn-based foods, which suggests that proso millet is a potentially good ingredient for the substitution of existing starches in producing low-GI and gluten-free products [8].

In recent years, there has been increasing interest in dietary fiber (DF), due to its health-promoting features [9]. Specifically, DF plays a crucial role in regulating the body’s metabolic processes of blood sugar and lipids, contributing to improved glycemic control and insulin sensitivity [10,11]. Additionally, incorporating DF into gluten-free bread enhances its nutritional value and improves its texture and shelf life [12,13]. For instance, guava pulp powder has been successfully employed to increase the antioxidant potential and complement the dietary fiber content of gluten-free bread without degrading its organoleptic qualities [14].

In our previous study, we obtained dietary fiber, termed F-DF, from defatted proso millet bran (DPB) through lactobacillus fermentation. F-DF displayed many notable physicochemical properties, including excellent water-holding capacity (WHC), oil-binding capacity (OHC), and water-swelling capacity (WS), as well as valuable adsorption characteristics, such as the adsorption of cholesterol, sodium cholate, and nitrous acid [15]. Cakes are widely consumed worldwide and known for their relatively dense yet tender texture and sweet taste [16]. Therefore, this study was designed with the objective of preparing dough and cake using proso millet flour (PF), fortified with F-DF, instead of conventional cake flour (WF), in order to analyze its effect on the rheological, microstructural, physicochemical, and functional characteristics, using conventional cake flour and gluten-free corn flour as controls; the obtained information can serve as a theoretical and practical foundation for the creation of gluten-free cake with advantageous properties.

## 2. Materials and Methods

### 2.1. Chemicals and Reagents

Proso millet (*Panicum miliaceum* L.), cornmeal, cake flour, and white sugar were purchased from Fugu County Tianmo Agricultural Products Co., Ltd. (Yulin, China). α-amylase (4000 u/g), papain (800,000 u/g), glucoamylase (100,000 u/g), pepsin (800,000 u/g), trypsin (400 U/L), glycolytic enzyme (10,000 U/L), and gallic acid were purchased from Shanghai Yuanye Biotechnology Co., Ltd. (Shanghai, China). 1,1-diphenyl-2-picrylhydrazyl free radical (DPPH) and 2,2′-azino-bis (3-ethylbenzothiazoline-6-sulfonic acid) (ABTS) were purchased from Shanghai Taitan Technology Co., Ltd. (Shanghai, China). Other reagents were of analytical grade.

### 2.2. Preparations of F-DF

The method of extracting DF from Fermented DPB was based on the AOAC enzyme gravimetric method, with minor modifications [17]. Fermented DPB was mixed with α-amylase at 95 °C and stirred continuously for 30 min. After the mixture cooled down to 45 °C, papain was added and the mixture was stirred continuously for another 30 min. Then, the pH of the mixture was adjusted to 4.5, glucosidase was introduced, the mixture was continuously stirred at 55 °C for 30 min, and then it was centrifuged at 3000× *g* for another 10 min. Subsequently, the supernatant was precipitated with 4 vol of ethanol (95%) to precipitate overnight, and then centrifuged to obtain DF. The obtained DF was freeze-dried and ground for later use.

### 2.3. Cake-Making Process

#### 2.3.1. Recipes

Table 1, which refers to Zhao et al. [18] with appropriate modifications, shows the recipe for the cakes.

#### 2.3.2. Batter Preparation and Cake Baking

The liquid egg, water, sugar, cake oil, and milk were mixed for 5 min using a mixing gadget. Then, the mixture of dry ingredients was sifted into the whipped egg mixture in three portions with a sieve, and mixed in for 10 min. The resulting mixture was poured into the prepared mold, placed in a baking oven, and baked at an oven temperature of 190 °C for 17 min.

### 2.4. Dough Properties Analysis

#### 2.4.1. Mixing and Pasting Properties of Dough

The determinations of the mixing and pasting behaviors of the various dough samples were carried out using mixolab according to the method of Gujral et al. [19]. The Chopin+ test method was used to test the mechanical properties of the cake flour dough. Phase 1: Hold at 30 °C for 8 min. Phase 2: Heat from 30 °C to 90 °C at a rate of 4 °C/min. Phase 3: Hold at 90 °C for 7 min. Phase 4: Cool to 50 °C at a rate of 4 °C/min. Phase 5: Hold at 50 °C for 5 min. The total test time was 45 min.

#### 2.4.2. Dynamic Rheological Properties of Dough

The rheological properties of the dough samples prepared in 2.4.1 were determined via dynamic rheometry, using a parallel plate system (40 mm diameter) at a gap of 1000 μm with a rheometer (DHR-1, Waters) to record the rheological energy. The shear rates ranged from 0.1 to 1000 s^−1^ according to the method published by Meng Niu et al. with minor modifications, steady shear experiments conducted at 25 °C, storage modulus (G′) and loss modulus (G″) measurements in the frequency range of 0.1–20 Hz, at a fixed pressure (1%), and 25 °C [20].

#### 2.4.3. Confocal Laser Scanning Microscopy (CLSM) of Dough

The microstructures of the dough samples were observed using confocal laser scanning microscopy (CLSM), and the experiments were carried out with reference to Silva et al. [21]. The dough samples prepared in 2.4.1 were post-stained with a solution of 0.25% (*w*/*w*) Fluorescein 5-isothiocyanate (FITC) and 0.025% Rhodamin B in water. CLSM observation conditions: FITC was observed at an excitation wavelength of 488 nm and an emission wavelength of 518 nm. Rhodamine was observed at an excitation wavelength of 568 nm and an emission wavelength of 625 nm.

### 2.5. Characterization of Cakes

#### 2.5.1. Analysis of Cake Properties

Ci 7600 was used to determine the color of the cake crumb and crust. L* indicates luminosity, with larger values indicating higher luminosity and smaller values indicating lower luminosity; a* and b* indicate different hue directions, with a+ indicating an increase in the red hue of the sample [22]. Furthermore, the color difference (ΔE) of the cakes was calculated as follows:

$$\Delta E=\sqrt{{\left(L^{*}-L_{0}^{*}\right)}^{2}+{\left(a^{*}-a_{0}^{*}\right)}^{2}+{\left(b^{*}-b_{0}^{*}\right)}^{2}}$$

where L_0_*, a_0_*, and b_0_* represent the color values of the control WF cake.

#### 2.5.2. Textural Analysis of Cakes

The measurement parameters were set as follows: TA/36R, speed of 3 mm/s before the test; 1 mm/s during and after the test; trigger force of 10 g, deformation of 30%, and compression dwell time of 20 s.

#### 2.5.3. Antioxidant Properties

The total phenolic contents (TPC), DPPH scavenging activity, and ABTS scavenging activity of the cakes were determined using the same method as in previous research methods [15].

$$DPPH\;scavenging\;activity\;\left(\%\right)=(1-\frac{A_{1}-A_{2}}{A_{0}})\times 100\%$$

$$ABTS\;scavenging\;activity\;\left(\%\right)=(1-\frac{A_{1}-A_{2}}{A_{0}})\times 100\%$$

Here, A_1_ is the absorbance of the sample (0.5 mL sample + 2.5 mL DPPH/ABTS solution), A_2_ is the absorbance of the control group (0.5 mL sample + 2.5 mL ethanol), and A_0_ is the absorbance of the blank group (0.5 mL ethanol + 2.5 mL DPPH/ABTS solution).

#### 2.5.4. Digestive Properties

The in vitro starch digestibility of each cake was determined according to the method described by Jia et al. [23], with minor modifications. A 0.5 g sample of the cake was suspended in 10 mL of sodium acetate buffer (0.25 M, pH = 5.2). Then, a trypsin solution with an enzyme activity of 400 U/L and a glycolytic enzyme solution with an enzyme activity of 10,000 U/L were added, and the test tube was placed at 37 °C and 170 r/min. The reaction was terminated by adding 4 mL of anhydrous ethanol to the supernatant. The glucose content in the supernatant of each digestion stage was determined using the 3,5-dinitrosalicylic acid (DNS) reagent method.

### 2.6. Statistical Analyses

All experiments were performed in triplicate. Statistical analyses were performed with GraphPad Prism.7. The data were analyzed with analysis of variance (ANOVA) and expressed as mean ± standard deviation (S.D.). *p* < 0.05 was considered to be statistically significant.

## 3. Results

### 3.1. Dough Properties

#### 3.1.1. Analysis of Mixing and Pasting Properties

Mixolab provides information that predicts the effects of mechanical changes and heating on protein, starch, and enzyme properties in a single test [24]. In theory, the farinograph indices of the CF, PF, and PF-DF dough samples are presented in Table 2. A cake’s water absorption (WA) is affected by multiple factors, including amylose, protein, damaged starch, and other ingredient factors in the flour [25,26]. The presence of PF significantly decreased the WA of the flours (*p* < 0.05), and there was no change in the WS of PF after the addition of F-DF, probably due to the lack of protein (gluten) in the PF, which has weak water absorption and hinders the formation of more gluten.

Dough development time (DDT) is a critical farinograph parameter that reflects the dough quality. A longer DDT means a stronger dough [27]. The DDT was 1.22 min for WF, 1.85 min for PF, and 2.20 min for PF-DF, demonstrating that the PF-DF flour can enhance gluten strength and flexibility. This result is similar to the powdery properties exhibited by Jasim Ahmed et al. [28]. In this study, it is noteworthy that the dough stability time (DS) of the PF dough showed an opposite trend with DDT. The primary factor is that PF is a gluten-free flour and lacks the protein to form a gluten network, which resulted in difficulty forming a stable gluten network [29].

The protein weakening degree of the PF dough increased more significantly compared to that of the WF dough, indicating that proso millet increased protein weakening, which was more pronounced with the addition of F-DF. This is outlined in the absence of gluten and protein network structure in PF dough, resulting in higher protein weakening degrees and decreased dough consistency. Similar results were reported by Yanrong Ma et al. [30] when different amounts of quinoa flour were supplemented with refined wheat flour in making crispy biscuits.

Starch gelatinization is an order-to-disorder transition that leads to the loss of birefringence, as well as the increase in swelling power and solubility [31]. During cooking, starch particles undergo gelatinization and partial solubilization, rendering them more accessible to digestive enzymes and, ultimately, enhancing the digestibility of starch [32]. The results presented in Table 2 demonstrate that CF exhibited significantly higher starch pasting qualities than PF, which has lower starch pasting characteristics and may partially impede starch digestion. This can lead to a decrease in postprandial blood glucose levels, rendering it suitable for consumption by special populations, such as people with diabetes. The textural characteristics, shelf life, and acceptance of items made from starch are all significantly impacted by starch regeneration [33]. The result indicates that the regeneration index of PF is significantly lower than that of CF. In contrast, the regeneration index of F-DF increased after adding dietary fiber, suggesting that proso millet is less prone to regeneration and aging, which can, to some extent, ensure the quality of food products with longer shelf lives.

#### 3.1.2. Rheological Properties

**Figure 1:**
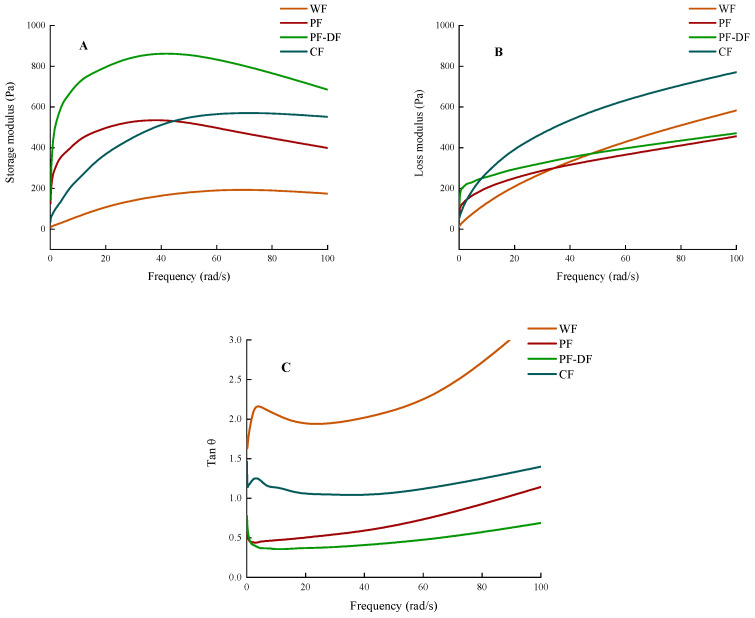
Rheological properties of doughs with different ingredients. (**A**) Energy storage moduli of WF, PF, PF-DF, and CF doughs; (**B**) loss moduli of WF, PF, PF-DF, and CF doughs; (**C**) loss angles of WF, PF, PF-DF, and CF doughs.

The rheological property of dough is a crucial to effectively predicting the quality of the final product [34,35]. Figure 1 illustrates the rheological properties of four different types of dough. The quality of bakery products is related to the rheological property of the dough, which, in turn, impacts the quality characteristics of the resulting bread, such as loaf volume and crumb cell structure [3,36]. The storage modulus (G′) and loss modulus (G″) sample oscillation curves of four dough samples are provided in Figure 1A, B. Both the G′ and G″ of the dough samples marginally increased with a rise in frequency at lower frequencies. Of the four samples measured, the PF-DF dough had the highest G′ and the second highest G″, after PF, as well as the smallest tan θ among all samples, which indicates that the addition of dietary fiber reduced the viscosity and fluidity of the dough. Overall, the proso millet doughs had good elasticity at low frequencies. However, as the frequency increased, the elasticity of the proso millet dough decreased, probably mainly due to the lack of gluten protein in the proso millet flour, which thus formed an unstable structure. Adding dietary fiber increases the dough’s elasticity and reduces its viscosity, likely because the gel-like substances in dietary fiber and starch form complexes through hydrogen bonds and van der Waals forces, affecting the rheological properties of the dough [19].

#### 3.1.3. Microstructure Analysis

**Figure 2:**
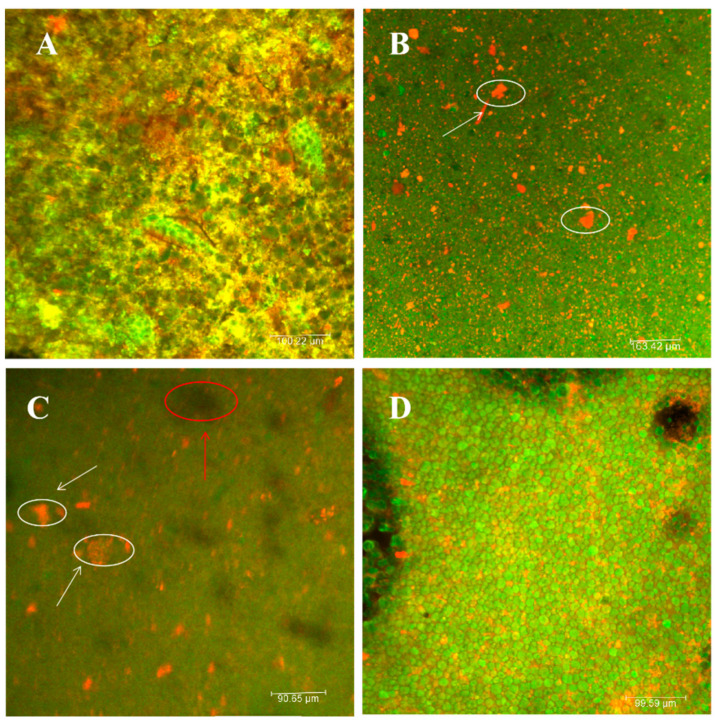
Confocal laser microstructures of cake dough made with different materials. (**A**) Confocal laser scanning microscopy of WF dough, (**B**) confocal laser microstructure of glutinous PF dough, (**C**) confocal laser microstructure of PF-DF dough, and (**D**) confocal laser microstructure of gluten-free CF control dough. The white circles represent protein and the red circles represent dietary fiber.

Dough samples were analyzed using CLSM, and all samples were post-labeled with a solution of 0.25% FITC and 0.025% Rhodamin B [21,37]. FITC preferentially stains starch green, and Rhodamin B preferentially stains protein red. The CLSM diagram of CF is shown in Figure 2A. With the proteins in the dough spread in a lamellar pattern and the starch in a punctate aggregated condition, the red proteins create a web-like spatial structure that encloses the green starch granules. Figure 2B shows the microstructure of the dough after replacing the CF with PF. The figure shows that the proteins in the dough started to be distributed from the original lamellar structure to the dotted structure, and the starch in it started to change, from the original dotted aggregated state to the dotted dispersed structure. This change in structure was mainly due to the lack of a formation of a stable reticulated spatial structure of the proteins. As can be seen from Figure 2C, the spatial distribution structures of both protein and starch after the addition of F-DF dietary fiber are basically similar to those of PF. In contrast, the lamellar structure of dietary fiber can be clearly seen in the figure (marked in red). In the gluten-free control group, shown in Figure 2D, the proteins in the cornmeal dough were distributed in scattered sheets, with spherical cornstarch granules embedded within them, and an extensive protein network was observed. These results suggested that the PF was without an obvious structural network, with starch randomly and loosely distributed within the protein matrix. Patino-Rodriguez et al. [38] observed that spaghetti made with unripe plantain flour similarly showed swollen starch granules that were loosely packed without a well-defined structural network.

### 3.2. Cake Properties

#### 3.2.1. Chroma Analysis of Crumb and Crust of Cakes

Color plays a crucial role in cakes as it, along with texture and aroma, greatly influences consumer preference. The colors of the crumb and crust of the cakes are shown in Table 3. Among the four groups of cakes, two groups of cakes made from proso millet had significantly lower crust color a* values than b* values, and positive b* values. Furthermore, this difference was more pronounced with adding F-DF to the PF, which may be influenced by protein content. Lower protein content reduces the likelihood of the Maillard reaction, resulting in fewer brown compounds [39,40]. The crumb’s color has a direct connection with the color of the ingredients, since the inside temperature does not rise above 100 °C without the occurrence of Maillard reactions [41]. Compared to the Control, the crumb color showed greater variation with proso millet, with considerable increases in a*, b*, and ΔE, and a significant decrease in L*, probably due to the yellow color of the proso millet itself.

#### 3.2.2. Analysis of Cake Textures

In order to explore the feasibility of proso millet flour and dietary fiber as raw materials in cake, and the effects of these additions on cake quality, the different cakes were characterized in terms of texture parameters, such as stiffness, extensibility, and strength. Table 4 summarizes the textural properties of the cake variations. Cake quality was adversely connected with an increase in cake hardness [42]. The gluten-free CF cake had the highest hardness value (*p* < 0.05), followed by the cake made with PF; the cake with WF presented the lowest hardness value, while with the addition of F-DF, no significant differences were seen compared to PF. At the same time, as the hardness of the cake increases, the chewiness of the cake increases accordingly, mainly because the chewiness of the product is positively related to the hardness and the increase in hardness leads to a rise in the chewiness of the product [43]. Similar results have been previously reported for gluten-free bread containing modified dietary fibers [12]. Adding PF and F-DF reduced the elasticity of the dough and increased its hardness, which had no significant effects on its viscosity, cohesion, and resilience, indicating that the whole proso millet flour with added F-DF had a lower sensory impact on the cake.

#### 3.2.3. Analysis of Antioxidant Properties

**Figure 3:**
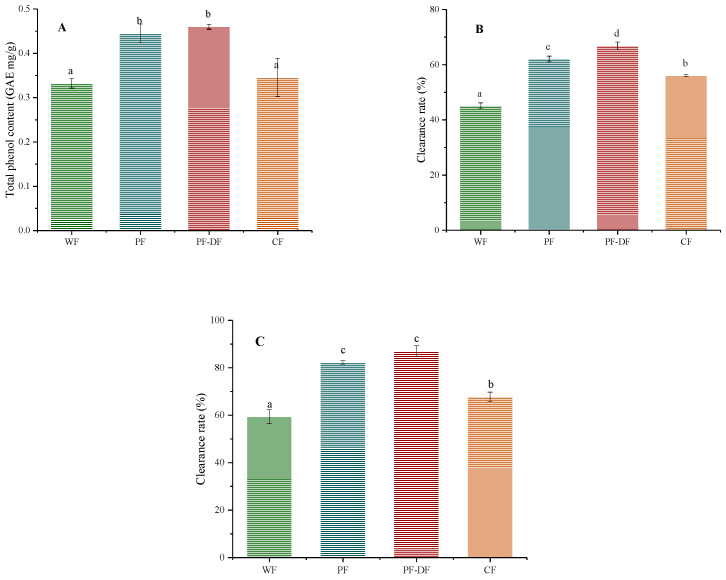
(**A**) The total phenolic contents of different cake extracts; (**B**) the DPPH free radical scavenging ability of different cake extracts; and (**C**) the ABTS free radical scavenging ability of different cake extracts. Data were represented as mean ± SD, *n* = 3; different lowercase letters on the bar charts indicate significant differences between different samples (*p* < 0.05).

In plants, polyphenols are common active secondary metabolites. Previous studies have shown that polyphenols have outstanding antioxidant capacity and biological activity. However, the usual reduction in bioactivity observed during the baking of enriched foods mainly results from the thermal degradation of the functional ingredients [15,44]. Figure 3A displays the TPC of four distinct cake extracts. As expected, PF and PF-DF showed high contents of total polyphenols (0.44 GAE mg/g; 0.46 GAE mg/g); these could be due to the difference in polyphenols contained between the raw materials. The antioxidant capacity, as assessed using ABTS and DPPH, was examined, and the results are presented in Figure 3. One of the finest methods for assessing antioxidant capability is the ABTS examination, which uses the radical cation (ABTS) derived from ABTS [44,45]. The antioxidant capacities and polyphenol contents in the cakes showed the same trend, and the order of the scavenging capacity of DPPH and ABTS in the cake extracts was PF-DF > PF > CF > WF, suggesting PF can improve the antioxidant capacity of cake over WF. Antioxidant capacity was further enhanced after the addition of dietary fiber. These results indicated that PF-based cakes had high polyphenol contents, improved the antioxidant activity of the cakes, and may play a preventive role in chronic diseases.

#### 3.2.4. Analysis of Digestive Properties

**Figure 4:**
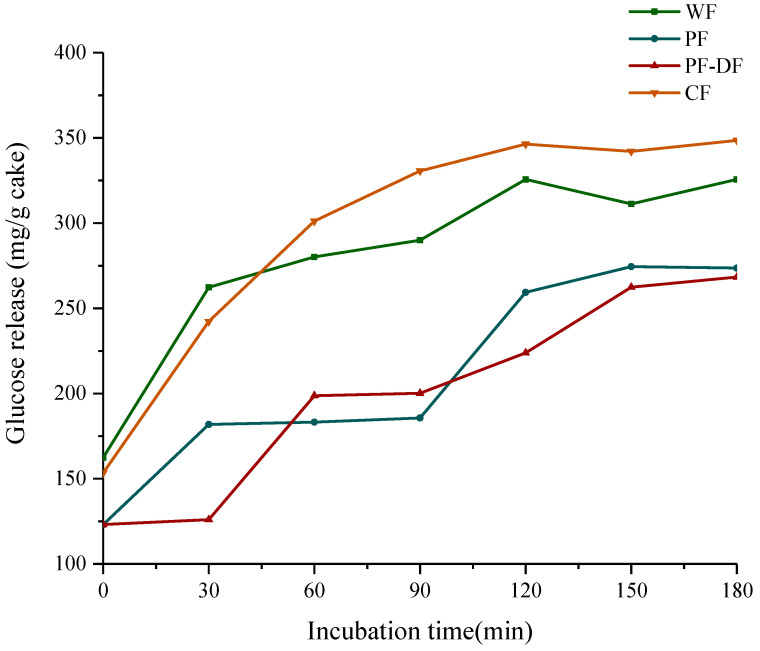
Glucose release curves during starch digestion of different cakes in vitro.

In vitro, simulated digestion can reflect the digestion and absorption of the substance [46]. In this work, the digestive properties of four different cakes were evaluated with in vitro digestion simulation experiments in order to evaluate the feasibility of replacing WF with PF to reduce the glycemic index of cakes. Figure 4 reports the glucose released during the 3 h period of intestinal digestion. The contents of the released glucose in the PF cake group were significantly lower than those in the WF and CF cake groups, and the glucose release was further reduced after adding dietary fiber, especially in 60–90 min; the glucose release gap between the PF cake and the other two cakes was the largest. The proportion of amylose and resistant starch in proso millet starch is high, which is not conducive to the digestion of starch granules, thereby reducing the release of glucose during cake digestion [47,48]. Furthermore, after adding the F-DF, the release of glucose during the digestion of the proso millet cake was further reduced. Dietary fibers tend to form high-viscosity gels upon hydration, forming a protective layer around the starch, thereby resulting in reduced interaction with α-amylases and slowing carbohydrate digestion [5,49].

## 4. Conclusions

The appropriateness of proso millet flour and fermented proso millet dietary fiber for baking cakes was examined, in order to increase the currently inadequate intake of dietary fiber in gluten-free diets. Although PF and F-DF hindered the formation of the gluten network structure, PF cakes accumulated polyphenols and enhanced antioxidant properties, in addition to decreasing glycemic index values. Notably, PF produced a poor effect on the cake’s texture, but maintained acceptable consumption qualities for consumers. Thus, the incorporation of proso millet in cakes not only provides better functional properties, but also improves their nutritional properties, especially for gluten-free products.

## Figures and Tables

**Table 1:** The basic recipes of the different cakes.

Ingredients	WF	PF	PF-DF	CF
Egg (g)	110	110	110	110
Cake flour (g)	50	-	-	-
Non-waxy proso millet flour (g)	-	10	9.4	-
Waxy proso millet flour (g)	-	40	37.6	-
Fermented proso millet dietary fiber flour (g)	-	-	3	-
Corn flour (g)	-	-	-	50
Sugar (g)	30	30	30	30
Salt (g)	0.5	0.5	0.5	0.5
Cake oil (g)	8	8	8	8
Water (mL)	10	10	10	10
Baking Powder (g)	0.5	0.5	0.5	0.5

WF—cake flour control group; PF—proso millet flour group; PF-DF—proso millet flour combined with fermented dietary fiber group; CF—gluten-free cornmeal control group.

**Table 2:** Effects of glutinous proso millet flour substituted for cake flour, and the addition of dietary fiber to glutinous proso millet flour, on flour quality characteristics.

	WF	PF	PF-DF	CF
C (%)	57.4 ^b^	50.5 ^a^	50.5 ^a^	-
Water absorption (%)	12.7 ^c^	12.1 ^b^	11.4 ^a^	-
Dough development time (min)	1.22 ^a^	1.85 ^b^	2.20 ^c^	-
Dough stability (min)	8.3 ^c^	5.9 ^b^	4.0 ^a^	-
Protein weakening degree (N/m)	0.495 ^c^	0.388 ^b^	0.341 ^a^	-
Pasting property of starch (N/m)	1.50 ^b^	1.37 ^a^	1.39 ^a^	-
Thermal stability of starch gelatinization (N/m)	−0.497 ^c^	−0.183 ^b^	−0.176 ^a^	-
Starch regeneration characteristic (N/m)	1.99 ^c^	0.70 ^a^	0.80 ^b^	-

Under the experimental conditions, the apparatus could not operate properly due to the high viscosity of the corn flour, resulting in no data for this group. Obtained data were represented as mean ± SD, *n* = 3; different lowercase letters in the same column indicate significant differences between different samples (*p* < 0.05).

**Table 3:** Chroma of cake crust and crumb.

Sample	Crust Color				Crumb Color			
	L*	a*	b*	ΔE	L*	a*	b*	ΔE
WF	52.95 ± 0.68 ^a^	21.19 ± 0.28 ^d^	36.24 ± 0.10 ^b^	-	83.78 ± 0.32 ^c^	0.39 ± 0.035 ^b^	31.32 ± 0.18 ^b^	-
PF	57.25 ± 0.54 ^c^	10.80 ± 0.25 ^b^	41.76 ± 0.0.31 ^c^	12.54 ± 0.1 ^b^	78.92 ± 0.21 ^b^	2.167 ± 0.021 ^c^	39.09 ± 0.15 ^d^	9.33 ± 0.07 ^b^
PF-DF	58.29 ± 1.40 ^c^	7.58 ± 0.27 ^a^	37.35 ± 0.56 ^b^	14.71 ± 0.32 ^a^	71.65 ± 0.49 ^a^	4.06 ± 0.21 ^d^	37.10 ± 0.77 ^c^	13.93 ± 0.77 ^a^
CF	53.97 ± 0.56 ^b^	18.33 ± 0.11 ^c^	33.26 ± 0.31 ^a^	4.29 ± 0.18 ^c^	83.57 ± 0.28 ^c^	−0.387 ± 0.060 ^a^	27.35 ± 0.50 ^a^	4.06 ± 0.49 ^c^

Data were represented as mean ± SD, *n* = 3; different lowercase letters in the same column indicate significant differences between different samples (*p* < 0.05).

**Table 4:** Texture characteristics of different cakes.

	WF	PF	PF-DF	CF
Hardness (g)	201.51 ± 5.64 ^a^	306.28 ± 5.06 ^b^	338.72 ± 16.44 ^b^	1875.81 ± 174.87 ^c^
Adhesiveness (g·s)	−0.36 ± 0.21 ^a^	0.11 ± 0.21 ^a^	−0.36 ± 0.21 ^a^	0.00 ± 0.00 ^a^
Springiness (-)	0.96 ± 0.20 ^a^	1.02 ± 0.02 ^a^	0.97 ± 0.02 ^a^	0.88 ± 0.02 ^a^
Cohesiveness (-)	0.83 ± 0.30 ^a^	0.82 ± 0.03 ^a^	0.82 ± 0.04 ^a^	0.88 ± 0.03 ^a^
Gumminess (g)	167.94 ± 5.16 ^a^	251.96 ± 10.44 ^a^	278.61 ± 15.87 ^b^	1647.64 ± 200.31 ^c^
Chewiness (g)	160.53 ± 5.05 ^a^	257.73 ± 37.02 ^b^	270.85 ± 18.07 ^b^	1450.58 ± 191.77 ^c^
Resilience (-)	0.46 ± 0.03 ^a^	0.46 ± 0.03 ^a^	0.46 ± 0.03 ^a^	0.47 ± 0.03 ^a^

Data were represented as mean ± SD, *n* = 3; different lowercase letters in the same row indicate significant differences between different samples (*p* < 0.05).

## Data Availability

Data are contained within the article.
